# Genetic polymorphisms (rs10636 and rs28366003) in metallothionein 2A increase breast cancer risk in Chinese Han population

**DOI:** 10.18632/aging.101177

**Published:** 2017-02-22

**Authors:** Di Liu, Meng Wang, Tian Tian, Xi-Jing Wang, Hua-Feng Kang, Tian-Bo Jin, Shu-Qun Zhang, Hai-Tao Guan, Peng-Tao Yang, Kang Liu, Xing-Han Liu, Peng Xu, Yi Zheng, Zhi-Jun Dai

**Affiliations:** ^1^ Department of Oncology, Second Affiliated Hospital of Xi'an Jiaotong University, Xi'an 710004, China; ^2^ National Engineering Research Center for Miniaturized Detection Systems, School of Life Sciences, Northwest University, Xi'an 710069, China

**Keywords:** metallothionein 2A, polymorphism, breast cancer

## Abstract

Genetic polymorphisms of *MT2A* are frequently observed in many different cancers. We performed this case-control study, including 459 breast cancer (BC) patients and 549 healthy controls from Northwest China, to evaluate the associations between two common MT2A polymorphisms (rs10636 and rs28366003) and BC risk. The MT2A polymorphisms were genotyped via Sequenom MassARRAY. The individuals with the rs28366003 A/G, A/G-G/G genotypes underwent a higher risk of BC (P<0.0001). And, the minor allele G of rs28366003 was related to an increased BC risk (*P*<0.0001). We also found a significantly increased BC risk with rs10636 polymorphism among homozygote and recessive models (*P*<0.05). Further subgroup analysis by clinical characteristics of BC patients showed that Scarff, Bloom and Richardson tumor grade (SBR) 1-2 have a higher expression of the minor allele of these two MT2A loci than SBR 3. Our results indicated that the rs10636 and rs28366003 polymorphisms in MT2A increased BC risk in Northwest Chinese Han population.

## INTRODUCTION

Breast cancer (BC) is the most frequent cancer among women, expecting to be 252,710 new BC cases and 40,610 death cases among American women in 2017 [1]. And, the incidence of BC increased dramatically in Asian population in recent years [2]. It is widely known that genetic factors contribute to BC susceptibility [3-7]. Recent studies showed the expression of the Metallothionein 2A (*MT2A*) gene increased in some human neoplasms [8-10]. And, it has been reported that the high expression of *MT2A* associated with the stage and prognosis of tumors [9, 11, 12]. MT2 belongs to metallothioneins (MT), which have four groups (MT1, MT2, MT3 and MT4 proteins) and are encoded by a family of genes located on chromosome 16q13 [13]. The expression of MT1 and MT2 are the most active isoforms in human cells [14]. And, the researchers found that MT1/MT2 may play an important role in tumors via several crucial mechanisms including modulating p53 zinc-dependent activity, inhibiting NF-κB signaling, and regulating the PIK3/AKT and Rb/E2F pathways [15-17]. The functional isoforms of MT2A mRNA transcript have been reported to express at the highest levels in breast tissues and is positively related to the cell proliferation and histological grade of BC [18].

Rs28366003 and rs10636 polymorphisms are the most common loci of *MT2A*, having been found to be associated with many different cancers. Rs28366003 locates in the core promoter region, while rs10636 polymorphism is in the 3′UTR region of the *MT2A* gene [17]. A recent study found that the single nucleotide polymorphism (SNP) rs28366003 stood apart from rs1610216 and rs10636 is significantly related to the laryngeal cancer risk in a Polish population [12]. Forma studied the association between *MT2A* (rs28366003, rs1610216, and rs10636) and the risk of prostate cancer. And only the rs28366003 SNP in *MT2A* was observed to be correlated with the prostate cancer risk in Polish population [19]. Similarly, Krzeslak's data suggested that the rs28366003 polymorphism in *MT2A* was related to the BC risk in a Polish population [13]. Although several poly-morphisms have been identified in *MT2A,* the function of its polymorphisms in BC not being fully understood [20]. In this study, we investigated and comprehensive-ly assessed the association between two SNPs of *MT2A* (rs10636 and rs28366003) and BC risk in Chinese Han population.

## RESULTS

### Characteristics of the study population

No significant difference was observed in the distribution of age, menopausal status, body mass index, and procreative times between the cases and controls (*P*＞0.05), which suggested that the cases and controls of this study were matched adequately on general characteristics (Table 1). The genotypic frequencies of the *MT2A* rs10636 and rs28366003 polymorphisms among the controls were in accord with HWE (*P* = 0.343 and *P* = 0.363, respectively).

In terms of genotype and allele distributions of *MT2A* polymorphisms, two polymorphisms in *MT2A* (rs10636 and rs28366003) showed positive associations with BC risk (Table 2). Rs10636 was identified to increase the BC risk in codominant and recessive models (C/C *vs*. G/G: OR = 1.86, 95% CI: 1.17- 2.94, *P* = 0.008; C/C *vs*. G/G+G/C: OR = 1.96, 95% CI: 1.26- 3.05, *P* = 0.003). In addition, the rs28366003 polymorphism showed an association with BC risk in codominant, dominant, recessive and overdominant models ( A/G *vs*. A/A, OR = 2.29, 95%CI = 1.53-3.45, *P* < 0.0001; A/G+G/G *vs*. A/A, OR = 2.66, 95% CI = 1.78-3.96, *P* < 0.0001; A/G *vs.* A/A+G/G, OR = 2.23, 95% CI = 1.48-3.35, *P* < 0.0001). And, the minor allele G of rs28366993 was also related to increasing BC risk (G *vs*. A, OR = 2.87, 95% CI = 1.97-4.2, *P* < 0.0001).

**Table 1:** The characteristics of breast cancer cases and cancer-free controls

Characteristics	Cases	Control	*P* value
Number	459	549	
Age (mean ± SD)	49.09±11.02	48.80±8.28	0.61
Age of menarche (mean ± SD)	14.37±1.57		
Menopausal status			
Premenopausal	237	267	0.376
Postmenopausal	222	282	
Body mass index (kg/m2) (mean ± SD)	23.06±2.92	22.45±2.53	0.274
Procreative times			
<2	242	298	0.657
≥2	217	251	
Tumor size			
<2 cm	152		
≥2 cm	307		
Lymph node involvement			
Negative	184		
Positive	275		
Histological grade			
SBR 1-2	244		
SBR 3	215		
Venous invasion			
None–little	292		
Moderate–severe	167		
Immunohistochemistry results			
ER	–	202	
	+	257	
PR	–	208	
	+	251	
Her-2	–	330	
	+	129	
Ki67	≥ 14%	294	
	< 14%	165	

ER: Estrogen receptor; PR: Progesterone receptor; Her-2: human epidermal growth factor receptor 2; SBR: Scarff, Bloom and Richardson tumor grade

**Table 2:** Association between MT2A polymorphisms (rs10636 and rs28366003) and breast cancer risk

Model	Genotype	Case(459)	Control(549)	OR (95% CI)	P-value
rs10636			HWE=0.343		
Codominant	G/G	241 (52.5%)	290 (52.8%)	1.00	
	G/C	164 (35.7%)	224 (40.8%)	0.88 (0.68-1.15)	0.35
	C/C	54 (11.8%)	35 (6.4%)	1.86 (1.17-2.94)	**0.008**
Dominant	G/G	241 (52.5%)	290 (52.8%)	1.00	
	G/C-C/C	218 (47.5%)	259 (47.2%)	1.01 (0.79-1.30)	0.92
Recessive	G/G-G/C	405 (88.2%)	514 (93.6%)	1.00	
	C/C	54 (11.8%)	35 (6.4%)	1.96 (1.26-3.05)	**0.003**
Overdominant	G/G-C/C	295 (64.3%)	325 (59.2%)	1.00	
	G/C	164 (35.7%)	224 (40.8%)	0.81 (0.62-1.04)	0.10
Allele	G	646 (%)	804 (%)	1.00	
	C	272 (%)	294 (%)	1.15 (0.95-1.40)	0.16
rs28366003			HWE=0.363		
Codominant	A/A	378 (%)	508 (%)	1.00	
	A/G	70 (%)	41 (%)	2.29 (1.53-3.45)	**<0.0001**
	G/G	11 (%)	0 (%)	-	-
Dominant	A/A	378 (%)	508 (%)	1.00	
	A/G-G/G	81 (%)	41 (%)	2.66 (1.78-3.96)	**<0.0001**
Recessive	A/A-A/G	447 (%)	547 (%)	1.00	
	G/G	11 (%)	0 (%)	-	-
Overdominant	A/A-G/G	390 (%)	510 (%)	1.00	
	A/G	70 (%)	41 (%)	2.23(1.48-3.35)	**0.0001**
Allele	A	826 (%)	1057 (%)	1.00	
	G	92 (%)	41 (%)	2.87 (1.97-4.20)	**<0.0001**

### MT 2A polymorphisms and the clinical features of breast cancer

We also analyzed the relationships between *MT2A* SNPs and clinical features of BC, including tumor size, lymph node metastasis, ER/PR/HER-2 status, his-tological grade, and venous invasion. As shown in Table 3, when the G/G genotype was used as a reference, there was no significant relation between the rs10636 polymorphism and clinical parameters except the histological grade (C/C *vs.* G/G: OR = 0.46, 95% CI = 0.29- 0.74, *P* = 0.001; G/C+C/C *vs*. G/G: OR = 0.63, 95% CI = 0.42- 0.93, *P* = 0.02). The same result was found in the analysis of the rs28366003 polymorphism and the histological grade of BC (G/G *vs*. A/A, OR = 0.52, 95% CI = 0.33- 0.82, *P* = 0.005, shown in Table 4).

**Table 3:** The associations between the MT-2A rs10636 polymorphism and clinical characteristics of BC patients

Variables	GG(%)	GC(%)	*p*	OR(95%CI)	CC(%)	*p*	OR(95%CI)	GC+CC (%)	*p*	OR(95%CI)
Tumor size										
<2CM	57(37.5)	54(35.5)			41(27)			95(62.5)		
≥2CM	98(31.9)	104(33.9)	0.63	1.12(0.71-1.78)	105(34.2)	0.11	1.49(0.92-2.42)	209(68.1)	0.23	1.2(0.85-1.92)
LN metastasis										
Negative	64(34.8)	65(35.3)			55(30)			120(65.2)		
Positive	84(30.5)	103(37.5)	0.41	1.2(0.77-1.89)	88(32)	0.41	1.22(0.76-1.95)	191(69.5)	0.34	1.2(0.82-1.8
ER										
Negative	77(38.1)	63(31.2)			62(30.7)			125(61.9)		
Positive	79(30.4)	91(35.4)	0.135	1.41(0.9-2.21)	87(33.9)	0.174	1.3(0.87-2.15)	178(69.3)	0.1	1.3(0.94-2.05)
PR										
Negative	90(43.3)	96(46.2)			22(10.5)			118(56.7)		
Positive	111(44.2)	106(42.2)	0.58	0.90(0.61-1.33)	34(13.5)	0.46	1.25(0.69-2.29)	140(55.8)	0.84	0.96(0.66-1.39)
Her-2										
Negative	100(30.3)	112(33.9)			118(35.8)			230(69.7)		
Positive	47(36.4)	44(34.1)	0.48	0.84(0.51-1.37)	38(29.5)	0.14	0.69(0.41-1.13)	82(63.6)	0.21	0.76(0.49-1.16)
Ki-67										
<50%	80(27.2)	85(28.9)			129(43.9)			214(72.8)		
≥50%	42(25.5)	47(28.5)	0.84	1.05 (0.63-1.77 )	76(46.1)	0.63	1.12 (0.7-1.79 )	123(74.5)	0.68	1.1 (0.71-1.69 )
Histological grade										
SBR 1-2	65(26.6)	85(34.8)			94(38.5)			179(73.4)		
SBR 3	79(36.7)	83(38.6)	0.34	0.8 (0.51-1.26 )	53(24.6)	0.001	0.46 (0.29-0.74 )	136(63.3)	0.02	0.63 (0.42-0.93 )
Venous Invasion										
Non-little	34(11.6)	64(21.9)			194(66.4)			258(88.4)		
Moderate-severe	24(14.4)	65(38.9)	0.25	1.44 (0.77-2.69 )	78(46.7)	0.06	0.57 (0.32-1.02 )	143(85.6)	0.4	0.79 (0.45-1.38 )

LN: Axillary lymph node; ER: Estrogen receptor; PR: Progesterone receptor; Her-2: human epidermal growth factor receptor 2; SBR: Scarff, Bloom and Richardson tumor grade

Similarly, no significant correlation was detected between the rs28366003 polymorphism and other clinical features.

## DISCUSSION

It was reported that MT acts as a regulator in cell proliferation, apoptosis, and differentiation, which imply that MT may involve in carcinogenesis of BC [9, 11, 21-24]. The biological effects of MT are connected with its physiochemical properties [25, 26]. Under the stress situation, MT regulates cell apoptosis, inhibits cell death and improves cell survival. Recent studies suggested the regulation of MT were completed by preventing oxidative progress and binding with apoptotic signal [27, 28], in which MT could suppress free- radial- induced oxidative damage of tissues and cells.

The dysfunction of *MT2A* might be linked with the zinc release blockage and the reduction of intracellular zinc concentrations, which resulted in an increased risk of oxidative damage, as well as abnormal breast cells genesis [25]. As few studies concern to the relationship between the polymorphisms of *MT2A* and BC, we performed this case-control study in a Chinese population. Krzeslak, et al. have reported an association between rs28366003 polymorphism and the risk of ductal BC, prostate cancer, and laryngeal cancer in a Polish population [12, 13, 19]. We observed significant differences in genotypes distribution and allele frequencies of two *MT2A* gene polymorphisms locus (rs10636 and rs28366003) between BC patients and control groups (*P* < 0.05). The *MT2A* SNP rs28366003 is an A/G substitution that is situated in the core promoter region of the *MT2A* gene sequence between the TATA box and transcription initiation site [13]. The transition may reduce the binding to the core promoter region, which is a nuclear molecule regulates *MT2A* gene transcription [29]. In this way, *MT2A* SNPs might be associated with functional changes, which imply that they may be involved in the interactions with the behavior of BC cell. Finally, the biological features of BC cells are gradually influenced. In addition, *MT2A* rs10636 polymorphism is located in the 3′ untranslated region, which implies that it may be involved in interactions with other nucleotide polymorphisms [29]. Our results suggest that both of the two *MT2A* SNPs rs10636 and rs28366003 significantly influence the susceptibility of BC.

As indicated by Ki-67 immunohistochemistry, *MT2A* is related to the proliferation of BC[25]. And, the down-regulation of *MT2A* arrests growth in MCF-7 cell lines also suggested the involvement of *MT2A* in the proliferation of BC [29, 30]. Moreover, it has been demonstrated that *MT2A* regulates endothelial cell migration through transcriptional regulation of the expression of vascular endothelial growth factor-c(VEGF-c) [31]. And it is believed that *MT2A* affects the histological differentiation grade in BC. The expression of *MT2A* mRNA in histological grade 3 tumors were higher than grade 1 and 2 tumors [20]. But in our study, BC patients with SBR 1-2 have a higher expression of the minor allele of MT2A SNPs loci than patients with SBR 3. So, we assume *MT2A* poly-morphisms may mitigate the aggressive behavior of BC cell. However, according to our results, there were no associations between ER/PR/HER-2 status, lymph node metastasis and *MT2A* polymorphisms (rs10636 and rs28366003).

In terms of the current study limitations, the sample size was inadequate for a stratified analysis and analysis of mix-type BC. Besides, we did not investigate other predisposing factors, including high-dose radiation exposure, alcohol consumption, and postmenopausal obesity. The further study should assess these factors as well for a more precise evaluation.

In conclusion, this study showed that *MT2A* polymorphisms rs10636 and rs28366003 are associated with BC risk in Chinese Han population. And, the relationship of *MT2A* polymorphisms and the histological grade may guide us to judge prognosis of BC. Further functional studies and large population-based prospective studies are needed to provide accurate evidence about the influence of *MT2A* variants on BC.

## MATERIALS AND METHODS

### Ethics statement

This study was approved by the Ethics Committee of the Second Affiliated Hospital of Xi'an Jiaotong university (Xi'an, China). The research protocol was completed according to the approved guidelines.

### Subjects

The consent of all the participants was obtained after they were informed that their blood samples would be used for research purpose. The blood samples of 459 Chinese women with sporadic BC (mean age: 49.09 ± 11.02 years) were selected from the Second Affiliated Hospital of Xi'an Jiaotong University, Shaanxi Province, China. In addition, 549 age- and sex- matched healthy individuals (mean age: 48.80 ± 8.28 years) without any history of autoimmune or malignant diseases formed the control group. All of the BC cases and controls were Han population and from Northwest China. All the patients were diagnosed by histology or pathology, as described in our previous studies [32]. The data of clinicopatho-logical characteristics of patients, including tumor size, clinical stages, lymph node metastasis, menopausal status, procreative times, estrogen receptor (ER) status, progesterone receptor (PR) status, and human epidermal growth factor receptor type2 (HER-2) status, were obtained from the patients' medical records (Table 1).

### DNA extraction and genotyping

The samples were contained in tubes coating with ethylene-diaminetetraacetic acid and were stored at −80°Cafter centrifugated until analysis. Genomic DNA was extracted from whole blood using the Universal Genomic DNA Extraction Kit Ver. 3.0 (TaKaRa Bio Inc., Shiga, Japan). DNA concentration was measured by spectrophotometry (DU530 UV/VIS spectro-photometer, Beckman Instruments, Fullerton, CA, USA). Two tag-SNPs (rs10636 and rs28366003) were selected in this study. A multiplexed SNP MassEXTEND assay was designed by Sequenom MassARRAY Assay Design 3.0 Software (Agena Bioscience, Inc., San Diego, CA, USA). SNP genotyping was performed by the Sequenom MassARRAY RS1000, and the primers were listed in Table 5. The data analyses were accomplished by Sequenom Type 4.0 [33].

**Table 4:** The associations between the MT-2A rs28366003 polymorphism and clinical characteristics of BC patients

Variable	GG(%)	GC(%)	*p*	OR(95%CI)	CC(%)	*p*	OR(95%CI)	GC+CC (%)	*p*	OR(95%CI)
Tumor size										
<2CM	57(37.5)	54(35.5)			41(27)			95(62.5)		
≥2CM	98(31.9)	104(33.9)	0.63	1.12(0.71-1.78)	105(34.2)	0.11	1.49(0.92-2.42)	209(68.1)	0.23	1.14(0.74-1.75)
LN metastasis										
Negative	45(24.5)	56(30.4)			83(45.1)			139(75.5)		
Positive	67(24.4)	78(28.4)	0.8	0.94(0.56-1.56)	130(47.3)	0.83	1.05 (0.66-1.68 )	208(75.6)	0.98	1.01(0.65-1.55)
ER										
Negative	70(34.7)	65(32.2)			67(33.2)			132(65.3)		
Positive	69(26.8)	88(34.2)	0.18	1.37(0.87-2.18)	100(39)	0.07	1.51 (0.96-2.39 )	188(73.2)	0.07	1.44(0.97-2.16)
PR										
Negative	55(26.4)	67(32.2)			86(41.3)			153(73.6)		
Positive	77(30.7)	81(32.2)	0.54	0.86(0.54-1.39 )	93(37.1)	0.26	0.77(0.49-1.22)	174(69.3)	0.32	0.81 (0.54-1.22)
Her-2										
Negative	71(21.5)	82(24.8)			177(53.6)			259(78.5)		
Positive	35(27.1)	33(25.6)	0.49	0.82(0.46-1.45)	61(47.3)	0.16	0.7(0.43-1.15)	94(72.9)	0.2	0.74(0.46-1.18)
Ki-67										
<14%	44(145)	105(35.7)			145(49.3)			250(85)		
≥14%	27(16.3)	64(38.8)	0.98	0.99(0.56-1.76)	74(44.8)	0.52	0.83(0.48-1.45)	138(83.6)	0.69	0.9(0.53-1.52)
Histological grade										
SBR 1-2	49(20)	34(13.9)			161(66)			195(79.9)		
SBR 3	57(26.5)	61(28.4)	0.13	1.54(0.88-2.72)	97(45.1)	0.005	0.52(0.33-0.82)	158(73.5)	0.1	0.79(0.45-1.08)
Venous Invasion										
None-little	94(32.2)	102(34.9)			96(32.9)			198(67.8)		
Moderate-severe	51(30.5)	57(34.1)	0.9	1.03(0.64-1.65)	59(35.3)	0.6	1.1(0.71-1.81)	116(69.5)	0.71	1.08(0.72-1.63)

LN: Axillary lymph node; ER: Estrogen receptor; PR: Progesterone receptor; Her-2: human epidermal growth factor receptor 2; SBR: Scarff, Bloom and Richardson tumor grade

**Table 5:** Primers used for this study

SNP_ID	1st-PCRP	2nd-PCRP	UEP_SEQ
rs10636	ACGTTGGATGAGAACGCGACTTCCACAAAC	ACGTTGGATGCATAGAAAAAGGAATATAGC	GACGGAATATAGCAAACGGTCA
rs28366003	GCCGCCTTACATCGCGGTTCAGGGAACTG	GAAGCAACCGCCCTTGGAGGAGGCGTGGT	AACTCAGGTCAACTGGATGCA

### Statistical analysis

All the statistical analyses were completed using the SPSS software package (version 20.0; SPSS Inc., Chicago, IL, USA). Hardy-Weinberg equilibrium (HWE) was examined by comparing expected and observed frequencies using Alrlquin 3.1 program (L. Excoffier, CMPG, University of Bern, Switzerland). The genotype frequencies of observed values were compared with expected values obtained from HWE theory (p^2^+2pq+q^2^=1; p is the frequency of the wild-type allele and q is the frequency of the variant allele). The calculation was performed by *χ^2^* test and the degree of freedom was 1 in the cases and controls. The significant difference in allele and genotype frequencies between cases and controls was determined by Pearson's χ^2^ test [34, 35]. And the cancer risk linked with alleles and genotypes was calculated with an odds ratio (OR) and 95% confidence interval (CI). We evaluated the risk in the codominant model (Aa *vs*. AA and aa *vs*. AA), dominant model (AA+ Aa *vs*. aa), recessive model (aa *vs*. Aa+AA), overdominant model (AA+ aa *vs*. Aa) and the allele model (a *vs*. A) respectively (A: the major allele, a: the minor allele). A two-sided P-value < 0.05 was considered statistically significant in all the tests.

## Abbreviations

MT2A: metallothioneins 2A
BC: breast cancer
SNP: single nucleotide polymorphism
OR: odds ratio
CI: confidence interval
HWE: Hardy-Weinberg equilibrium
ER: Estrogen receptor
PR: Progesterone receptor
Her-2: human epidermal growth factor receptor 2
